# Central nervous system relapse of an extranodal natural killer/T‐cell lymphoma

**DOI:** 10.1002/jha2.1025

**Published:** 2024-10-04

**Authors:** Radu Chiriac, Lucile Baseggio, Camille Golfier

**Affiliations:** ^1^ Hospices Civils de Lyon Centre Hospitalier Lyon Sud Laboratoire d'hématologie Pierre‐Benite France; ^2^ Hospices Civils de Lyon Centre Hospitalier Lyon Sud Hématologie clinique Pierre‐Benite France

**Keywords:** central nervous system, cytology, extranodal NK/T‐cell lymphoma

1

A man in his 50s presented with rapid neurological decline and sudden onset of facial diplegia, occurring six months after receiving intensified therapy with autologous stem cell transplantation for stage IV extranodal natural killer (NK)/T‐cell lymphoma, nasal type (ENKTCL), which had been diagnosed 1 year earlier. Central nervous system (CNS) prophylaxis with high‐dose methotrexate (HD‐MTX) was initially administered.

On admission, blood work revealed no circulating lymphoma cells. Plasma Epstein–Barr virus DNA concentration was 830,000 copies/mL (reference range: 180–500 copies/mL). Brain and spine magnetic resonance imaging (MRIs) were normal. A lumbar puncture revealed a cerebrospinal fluid (CSF) cytospin preparation with monomorphic medium to large lymphomatous cells exhibiting irregular nuclear contours and variably condensed chromatin, along with scant to moderate cytoplasm containing azurophilic granules (Figure 1A). Flow cytometry of the CSF confirmed an aberrant NK/T‐cell phenotype: CD3‐, CD5‐, CD2+, CD7+, CD57+, and CD45RO+ (Figure 1B). Next‐generation sequencing of the CSF revealed mutations in *BCOR* (VAF 74%), *TP53* (VAF 76%), *DDX3X* (VAF 63%), and *JAK3* (VAF 34%), the same mutations as in the initial biopsy. A CNS leptomeningeal relapse of known ENKTCL was confirmed. Pembrolizumab and HD‐MTX were subsequently initiated, resulting in symptom regression.

**FIGURE 1:**
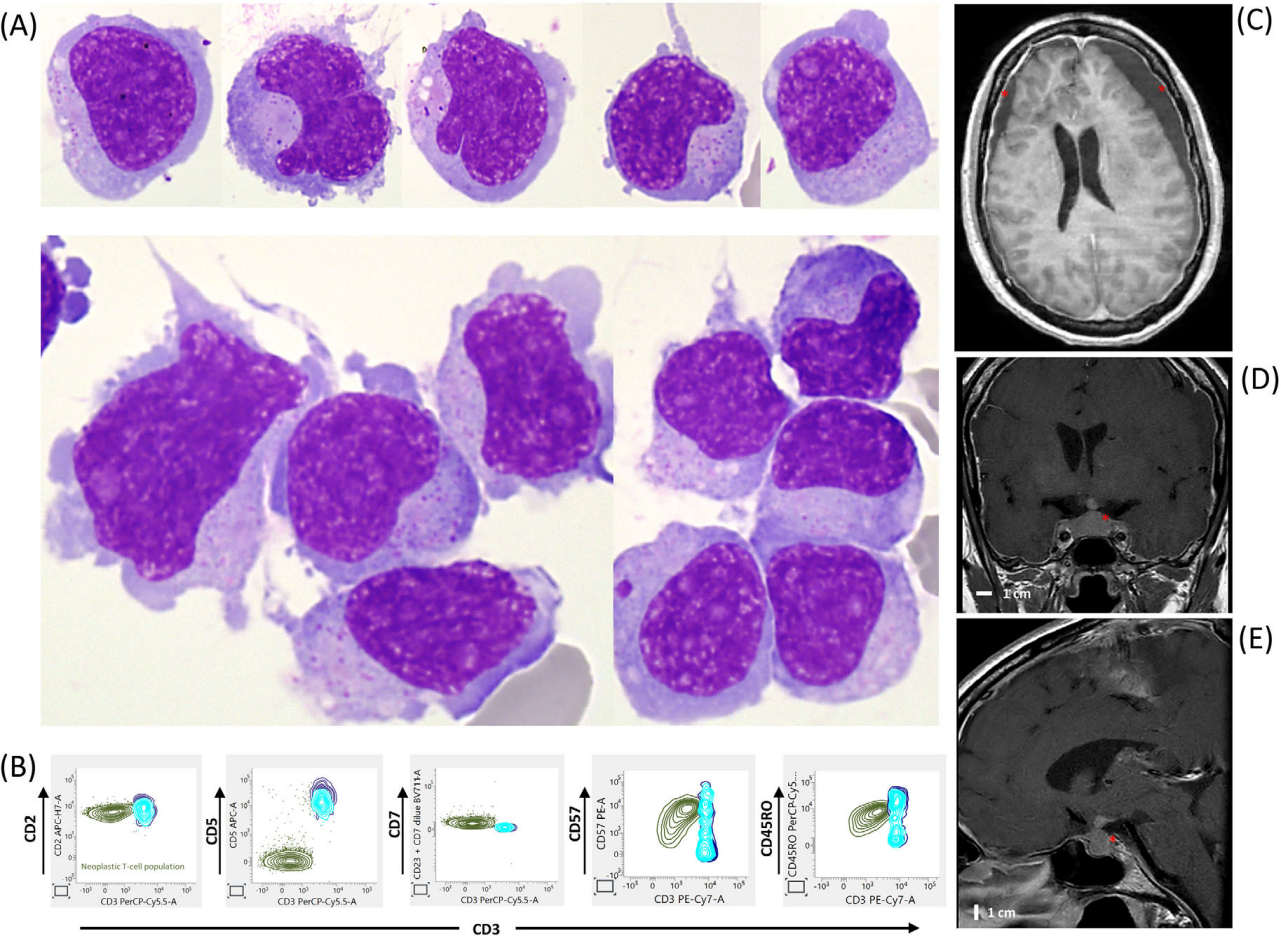
Panel (A) May‐Grünwald Giemsa stain, 1000x magnification, showing atypical lymphomatous cells. Panel (B) Flow cytometry profile showing CD3‐, CD5‐, CD2+, CD7+, CD57+, and CD45RO+ T‐cells. Panel (C) Axial section of a gadolinium‐enhanced magnetic resonance imaging (MRI) brain scan showing subdural detachment (asterisk). Panel (D) Coronal section, T1 TSE with gadolinium, MRI brain scan showing thickening of the pituitary gland (asterisk). Panel (E) Sagittal section, T1 TSE with gadolinium, MRI brain scan showing thickening of the pituitary stalk (asterisk).

After the second cycle, the patient presented with fatigue, muscle weakness, mood changes, and decreased vision associated with difficulty reading. There was a complete resolution of the initially described facial paralysis, and no lymphoma involvement was noted. Brain MRI revealed bilateral fronto‐parietal subdural detachment (Figure 1C, asterisk) suggestive of a subacute subdural hematoma, along with thickening of the pituitary gland (20 mm in width and 14 mm in height) (Figure 1D, asterisk) and nodular thickening of the pituitary stalk (6 mm) (Figure 1E, asterisk). Homogeneous enhancement of the pituitary gland and stalk after gadolinium injection indicated hypophysitis, with the pituitary stalk exerting a mass effect on the optic chiasm. No diffusion‐weighted hyperintensity of the pituitary gland was observed. Deficiencies in adrenocorticotropic and thyroid‐stimulating hormones were noted. Intravenous high‐dose hydrocortisone was initiated, followed by oral hydrocortisone in a progressively decreasing dose to address this episode of anti‐PD‐1 therapy‐induced hypophysitis.

However, the patient passed away one week later due to refractory status epilepticus, which was likely multifactorial. Possible contributing factors include subdural hematomas (with acute mass effect noted at a later stage), pembrolizumab‐induced neurotoxicity, and metabolic disturbances secondary to hypophysitis (such as severe hyponatremia and hypoglycemia).

ENKTCL is a rare non‐Hodgkin's lymphoma that rarely spreads to the CNS. Stage III/IV disease significantly increases the risk of CNS involvement in this category of patients [1]. Treating progression or relapse in the CNS is a significant challenge. Although HD‐MTX can be effective in achieving a CNS response, systemic disease often remains a major cause of mortality, as demonstrated in this case. Aside from focusing on the side effects of PD‐1 inhibitors, hypophysitis, though rarely encountered, can occur at any point during treatment and regardless of the type of cancer [2] as evidenced in this scenario, complicating the progression.

## AUTHOR CONTRIBUTIONS

Radu Chiriac and Lucile Baseggio wrote the manuscript and conducted the cytological and immunological examinations. Camille Golfier followed the patient and supplied the patient's information. All authors contributed to the final manuscript.

## CONFLICT OF INTEREST STATEMENT

The authors declare no conflict of interest

## FUNDING INFORMATION

The authors received no specific funding for this work.

## ETHICS STATEMENT

This manuscript respects the ethical policy of CHU Lyon for the treatment of human research participants.

## PATIENT CONSENT STATEMENT

No patient‐identifying data were used. The authors did not obtain written informed consent from the patient but the patient did not object to his data being used for research purposes (as required by the ethical policy of CHU Lyon).

## CLINICAL TRIAL REGISTRATION

The authors have confirmed clinical trial registration is not needed for this submission.

## Data Availability

Data sharing is not applicable to this article as no new data were created or analyzed in this study.
